# Association of Sex Differences with Mortality and Organ Dysfunction in Patients with Sepsis and Septic Shock

**DOI:** 10.3390/jpm13050836

**Published:** 2023-05-15

**Authors:** Caspar Mewes, Julius Runzheimer, Carolin Böhnke, Benedikt Büttner, José Hinz, Michael Quintel, Ashham Mansur

**Affiliations:** 1 Department of Anesthesiology, University Medical Center Goettingen, 37075 Goettingen, Germany; carolin.bhnke@stud.uni-goettingen.de (C.B.); benedikt.buettner@med.uni-goettingen.de (B.B.); mquintel@med.uni-goettingen.de (M.Q.); ashham.mansur@med.uni-goettingen.de (A.M.); 2 Center of Anesthesiology and Intensive Care Medicine, University Medical Center Hamburg-Eppendorf, 20251 Hamburg, Germany; 3 Department of Neurology and Neurophysiology, University Medical Center Freiburg, 79106 Freiburg, Germany; 4 Department of Anesthesiology and Intensive Care Medicine, Klinikum Region Hannover, 30459 Hannover, Germany; jose.hinz@krh.eu; 5 Department of Anesthesiology, Asklepios Hospitals Schildautal, 38723 Seesen, Germany

**Keywords:** sepsis, septic shock, sex, sex differences, sepsis mortality, organ dysfunction, critical illness

## Abstract

Background: Despite recent advances in the clinical management and understanding of sepsis and septic shock, these complex clinical syndromes continue to have high mortality rates. The effect of sex on these diseases’ mortality, clinical presentation and morbidity remains controversial. This study aimed to investigate the association of sex with mortality and organ dysfunction in patients with sepsis and septic shock. Methods: Prospectively enrolled patients with clinically defined sepsis and septic shock in three intensive care units at University Medical Center Göttingen, Germany, were investigated. The primary outcomes were 28- and 90-day mortality, while the secondary endpoints included the evaluation of organ dysfunction as measured by clinical scores and laboratory parameters. Results: A total of 737 septic patients were enrolled, including 373 in septic shock, 484 males, and 253 females. No significant differences in 28- and 90-day mortality were observed in the cohort. However, men with sepsis had significantly higher SOFA scores, SOFA respiratory and renal subscores, bilirubin and creatinine values, and lower weight-adapted urine outputs, indicating higher organ dysfunction compared to women. Conclusions: Our findings revealed notable differences in organ dysfunction between male and female patients, with males exhibiting more pronounced dysfunction across multiple clinical indicators. These results highlight the potential influence of sex on sepsis disease severity and suggest the need for tailored approaches in sepsis management according to patient sex.

## 1. Introduction

Sepsis and septic shock are complex life-threatening clinical syndromes that continue to pose significant challenges for clinicians and researchers worldwide. They are interdisciplinary pathologies that affect almost every department in the hospital and not only cause significant in-hospital mortality, associated long-term morbidity and reduced quality of life for patients concerned but also major expenses for public healthcare systems [1,2,3]. Despite advances in the understanding and clinical management of sepsis in recent years, mortality rates remain high, ranging from 25 to 30% in sepsis to over 50% in septic shock cases [4,5,6,7].

Recent research has suggested that sex-specific differences may play a role in the pathophysiology, clinical presentation and outcomes of sepsis, with potential implications for clinical management and treatment strategies. However, the findings were not consistent across all studies.

Several studies have reported that the mortality of sepsis and septic shock was significantly lower in female compared to male patients [8,9,10,11]. Furthermore, women were less likely to experience organ dysfunction and had a lower risk of developing sepsis-related complications such as acute respiratory distress syndrome (ARDS) [9,10].

However, other studies have found conflicting results reporting no significant difference in mortality between men and women or even increased mortality among women [12,13]. These controversial findings do not necessarily contradict the idea that sex may influence the pathophysiology of sepsis. Rather, they suggest that sex-specific differences in sepsis mortality may not be consistently observed across all patient populations or settings and need to be further investigated and confirmed in independent sepsis cohorts with precisely defined inclusion criteria.

Understanding the association of sex differences with mortality and organ dysfunction in patients with sepsis and septic shock remains a fundamental area of ongoing research. It may help to identify potential sex-specific prognostic risk factors for poor outcomes, which could be used to improve clinical management and develop risk-adjusted individualized treatment strategies. Moreover, insights into the underlying biological and molecular mechanisms—such as sex-specific modulations of the host immune response and the immunoregulatory role of sex hormones such as estrogen and testosterone—could lead to new approaches for preventing or treating critical conditions like sepsis and septic shock.

## 2. Materials and Methods

The present study was performed at the University Medical Center Göttingen, Germany. All investigations were approved under the ethical project identification code 1/15/12 by the institutional ethics committee of the University of Göttingen, Germany. The study was performed in accordance with the provisions, relevant guidelines and regulations of the Declaration of Helsinki. Written informed consent was obtained from all patients or their legal representatives.

This study was performed in accordance with the STROBE statement guidelines for reporting observational studies [14].

### 2.1. Patient Enrollment

This single-center investigation included a prospective cohort of 737 patients with sepsis, of which 373 met the International Consensus Sepsis-3 definition criteria for septic shock. The cohort was originally used to prospectively investigate the association of genetic variants and clinical characteristics with mortality, disease severity and the clinical phenotype of sepsis and septic shock. Patient enrollment occurred between 2012 and 2019 in three surgical ICUs at the University Medical Center Göttingen, Germany. All patients of these ICUs were screened daily for clinically defined sepsis in accordance with the latest guidelines and definitions [1,15]. Patients who met the eligibility criteria were added to the study database, GENOSEP, and monitored for 28 days with daily data collection, and their mortality status was tracked for 90 days through individual telephone follow-up or written requests from the local registry. None of the patients were lost at the time of follow-up. Non-eligible patients were excluded from the study according to the previously defined exclusion criteria [16,17,18,19,20,21]:

- Less than 18 years of age;
- Pregnancy and/or breastfeeding;
- Therapy with immunosuppressive drugs and/or chemotherapy within six months prior to enrollment;
- Myocardial infarction within six weeks before recruitment;
- Chronic heart failure, classified as New York Heart Association stage IV;
- Human immunodeficiency virus (HIV) infection and/or hepatitis B/C infection;
- End-stage incurable disease;
- Persistent vegetative state (apallic syndrome);
- “Do Not Treat (DNT)” or “Do Not Resuscitate” (DNR) order;
- Participation in interventional studies and family member of a study-site employee.

Eligible patients were dichotomized according to their sex into a male and female group.

### 2.2. Data Collection

All clinical and patient baseline data were generated from the electronic patient record system (IntelliSpace Critical Care and Anesthesia (ICCA), Philips Healthcare, Andover, MA, USA) using standardized clinical report forms (CRFs).

Recorded data included the patient baseline characteristics, such as basic characteristics (age, body mass index) and the disease severity or clinical condition at the time of enrollment (initial Sequential Organ Failure Assessment (SOFA) score, Acute Physiology and Chronic Health Evaluation (APACHE II) score, necessity of vasopressors, mechanical ventilation or renal replacement therapy). Patients’ recent surgical history (no surgery, elective or emergency surgery), as well as a primary site of infection (pulmonary, abdominal, bone or soft tissue, surgical wound, urogenital or primary bacteremia), were gathered. Additionally, common preexisting comorbidities, such as arterial hypertension, chronic obstructive pulmonary disease (COPD), bronchial asthma, renal dysfunction, non-insulin-dependent and insulin-dependent diabetes mellitus (NIDDM, IDDM), chronic liver disease, a history of myocardial infarction, stroke or cancer, and common long-term medication, including statins, beta-blockers, ACE inhibitors, bronchodilators, diuretics and anticoagulation, were recorded.

The relevant clinical parameters of disease severity were collected for a maximum duration of 28 days after sepsis onset, excluding patients that left the ICU or were earlier deceased. The disease severity parameters included the following: the SOFA and organ-specific SOFA scores (respiratory, coagulation, liver, cardiovascular, central nervous system, renal); days in septic shock, ICU and hospital length of stay; inflammatory values (leukocyte count, C-reactive protein, procalcitonin); respiratory values (total ventilated days, percentage of patients with mechanical ventilation, percentage of ventilated days during observation); coagulation (thrombocyte count); liver values (Bilirubin, aspartate transaminase (AST), alanine transaminase (ALT)); cardiovascular values (total days with vasopressor use, percentage of patients with vasopressor treatment, percentage of days with vasopressor treatment during observation); Glasgow Coma Score (GCS); and renal values (creatinine, total urine output per day, urine output per day per kilogram, total number of days with renal replacement therapy, percentage of patients with renal replacement therapy, percentage of renal replacement therapy during observation).

The primary outcome was 28- and 90-day mortality among men and women with sepsis and septic shock. The disease severity parameters served as secondary outcome variables.

### 2.3. Statistical Analysis

The categorical variables are presented as absolute numbers or percentages, while the continuous variables appear as the mean ± standard deviation or median and interquartile ranges, where applicable.

The Mann–Whitney U test was used to compare the continuous variables, whereas Pearson’s chi-square test or the two-sided Fisher’s exact test served to compare the discrete variables. The log-rank test was applied for Kaplan-Meier survival analyses.

Multivariate Cox proportional hazards regression analysis for the time until death was performed and adjusted for the relevant potential confounders that differed between the two groups at baseline. Hazard ratios (HRs) and associated 95%-confidence intervals (Cis) were calculated for 28- and 90-day mortality.

STATISTICA 13 software (version 13.0, StatSoft, Tulsa, OK, USA) was used for all statistical analyses. For the presented data, a *p*-value < 0.05 was considered statistically significant.

## 3. Results

### 3.1. Demographics and Patient Baseline Characteristics

A total of 737 patients were enrolled in the study, of which 484 were male and 253 were of female sex. None of the patients were lost at the time of the follow-up.

Table 1 presents the patient baseline characteristics stratified by sex. The average age was 63 ± 15 years, and the majority of patients were mechanically ventilated (87%) and received vasopressors (70%) at the time of sepsis onset. A total of 373 patients were in septic shock.

In the studied cohort, male patients presented a higher body mass index at enrollment (28 ± 7) compared to female patients (27 ± 7; *p* = 0.0062). Furthermore, male patients appeared to have NIDDM more often (10%) than female patients (5%; *p* = 0.0206) and more frequently received statins (26% vs. 19%; *p* = 0.0234) and ACE inhibitors (32% vs. 23%; *p* = 0.0097) as long-term medications at baseline.

There were no significant differences in severity, recent surgical history or the site of infection between the two groups at sepsis onset.

### 3.2. Survival Analyses

**Figure 1:**
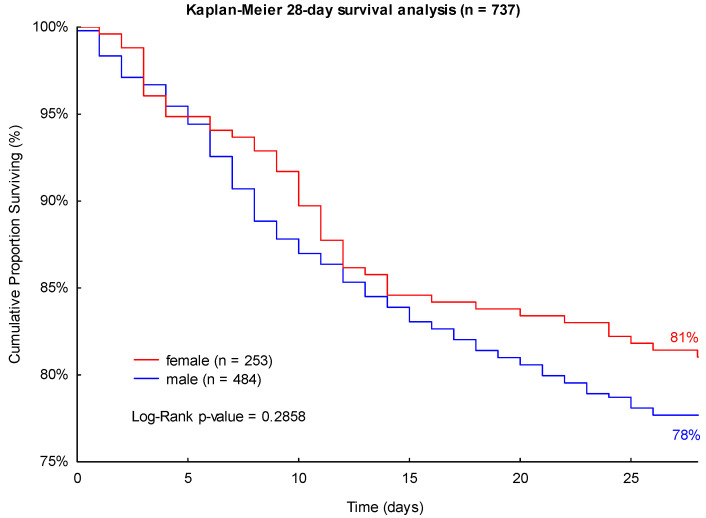
Kaplan-Meier 28-day survival analysis of patients with sepsis with regard to sex.

**Figure 2:**
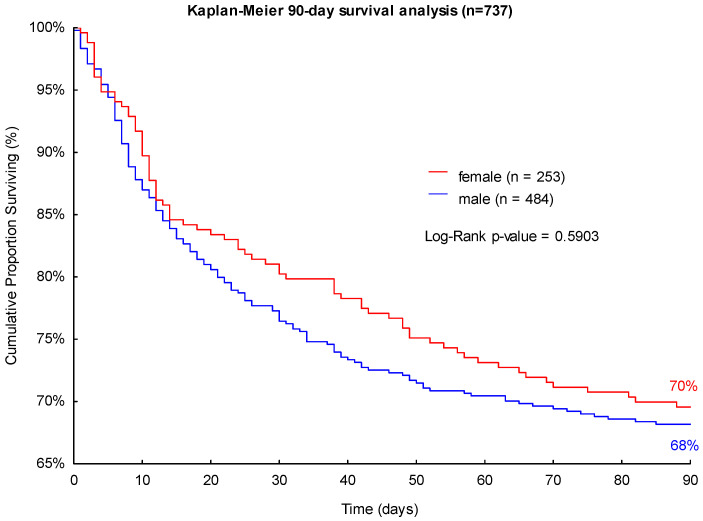
Kaplan-Meier 90-day survival analysis of patients with sepsis with regard to sex.

The observed 28- and 90-day mortality rates were higher in male patients (22% and 32%, respectively) than in female patients with sepsis (19% and 30%, respectively) in the studied cohort (see Figure 1 and Figure 2). However, the differences in mortality did not have statistical significance in the performed log-rank Kaplan-Meier survival analyses (*p* = 0.2858 and *p* = 0.5903; see Figure 1 and Figure 2).

**Figure 3:**
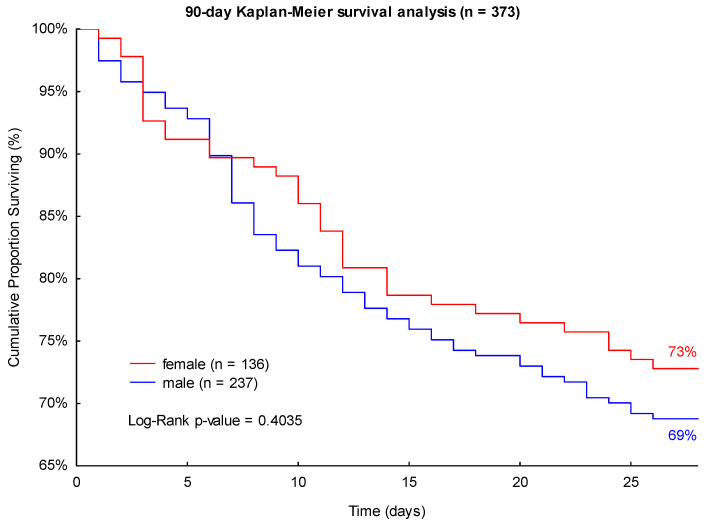
Kaplan-Meier 28-day survival analysis of patients with septic shock with regard to sex.

**Figure 4:**
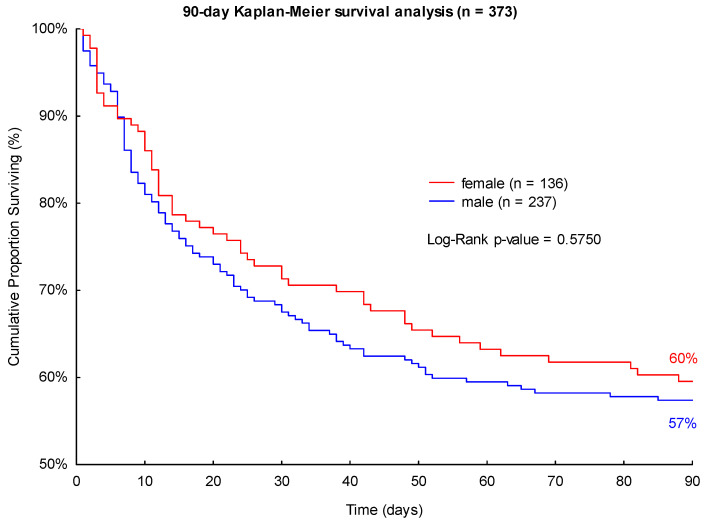
Kaplan-Meier 90-day survival analysis of patients with septic shock with regard to sex.

Accordingly, male patients presented a higher 28- and 90-day mortality in septic shock (31% and 43%) compared to female patients (27% and 40%; see Figure 3 and Figure 4). Neither of these findings was significant in the log-rank Kaplan-Meier survival analyses (*p* = 0.4035 and *p* = 0.5750, see Figure 3 and Figure 4).

### 3.3. Disease Severity Analysis

The results of the disease severity analyses of patients with sepsis and septic shock stratified by sex can be obtained from Table 2 and Table 3.

During the course of disease in sepsis, male patients presented significantly higher average SOFA scores (7.4 ± 3.6) than female patients (6.9 ± 3.8; *p* = 0.0265), higher organ-specific respiratory (2.0 ± 0.8 vs. 1.9 ± 0.8; *p* = 0.0091) and renal (0.3 vs. 0; *p* = < 0.001) SOFA subscores, as well as higher values of serum bilirubin (0.7 vs. 0.5; *p* = < 0.001), serum creatinine (1.4 ± 1 vs. 0.9 ± 0.6; *p* = < 0.001) and a lower urine output per kilogram per day (1.4 ± 0.7 vs. 1.7 ± 0.9; *p* = < 0.001, see Table 2).

Similar results were observed in patients with septic shock (s. Table 3). In this subcohort, male patients appeared to have significantly higher SOFA renal subscores (0.8 vs. 0.3; *p* = 0.0021), serum creatinine (1.6 ± 1 vs. 1.1 ± 0.7; *p* = < 0.001) and a lower urine output per kilogram per day (1.3 ± 0.8 vs. 1.5 ± 0.9; *p* = 0.0326) compared to the female study participants, accordingly.

### 3.4. Multivariate Cox Proportional Hazards Regression Analysis

We chose to input the sex, age and relevant variables from the patient baseline characteristics (BMI, NIDDM, statins and ACE inhibitor) into the multivariate Cox proportional hazards regression model (see Table 4 and Table 5). It revealed no significant relations between the male sex and 28- or 90-day mortality, neither in patients with sepsis (HR: 1.25, 95%-CI: 0.89–1.76, *p* = 0.2012 for 28-day mortality; HR: 1.09, 95%-CI: 0.83–1.44, *p* = 0.5236 for 90-day mortality) nor patients with septic shock (HR: 1.25, 95%-CI: 0.84–1.87, *p* = 0.2699 for 28-day mortality; HR: 1.15, 95%-CI: 0.82–1.6, *p* = 0.4142 for 90-day mortality). Except for age (HR: 1.04, 95%-CI: 1.02–1.05, *p* = < 0.001 for patients with sepsis; HR: 1.03, 95%-CI: 1.01–1.04, *p* = < 0.001 for patients with septic shock), none of the other variables were observed to have a significant association with the 28- or 90-day mortality in sepsis or septic shock in the multivariate model.

## 4. Discussion

The impact of sex on sepsis and septic shock is not fully understood, and the results from other cohorts remain controversial. This study aimed to explore the association of sex with mortality and organ dysfunction in a large, clearly defined, prospective single-center cohort of patients with sepsis and septic shock.

Our observational cohort study found no significant differences in 28- and 90-day mortality between male and female study participants. However, men showed significantly higher SOFA scores, including SOFA respiratory and renal subscores, as well as elevated serum bilirubin and creatinine values, and a lower urine output per kilogram per day, suggesting higher organ dysfunction compared to women with sepsis. Similarly, male patients with septic shock had higher SOFA renal subscores and serum creatinine levels and a significantly lower urine output per kilogram per day compared to female patients. Thus, despite the absence of significant differences in mortality between both sexes, our study revealed a significantly increased organ dysfunction, as measured by the organ-specific SOFA scores, including the SOFA respiratory and renal subscores, as well as elevated levels of bilirubin and creatinine in male patients.

Our findings are consistent with some of the previous studies, which have reported no significant differences in mortality between male and female patients with sepsis [11,22,23]. For instance, a post hoc analysis of 3902 patients from 24 participating medical and surgical ICUs in Italy by Sakr et al. (2013) reported a similar ICU mortality in men and women (20.1% vs. 19.8%) [11]. Another study, by van Vught et al. (2017), also reported no differences in the 90-day mortality and mortality up to 1 year after an ICU admission between men and women in 1533 patients with sepsis [23].

However, our findings contrast with other studies that have reported better outcomes in female patients with sepsis [10,24]. For example, Xu et al. (2019) conducted a retrospective analysis of 6134 adult patients with sepsis and found that female patients had lower rates of 1-year, 90-day and in-hospital mortality, as well as shorter hospital stays than male patients [10]. Similarly, a large prospective cohort study by Thompson et al. (2022), involving 12,912 sepsis hospitalizations in Australia, reported that female patients with sepsis had lower 1-year mortality rates and shorter hospital and ICU stays than male patients [24]. To the best of our knowledge, a comprehensive evaluation of mortality and organ dysfunction differences, specifically between male and female patients during septic shock, according to the currently valid definitions, has not been documented in the existing literature.

Our findings of sex-specific differences in disease severity of sepsis and septic shock, specifically in the respiratory and renal SOFA subscores, serum creatinine levels and urine output, align with existing studies. A study of 18,757 ICU patients conducted by Pietropaoli et al. (2010) revealed that female patients were less likely to receive invasive mechanical ventilation or hemodialysis catheters compared to men [13]. Furthermore, Modra et al. (2022) reported in a systematic review and meta-analysis of 545,538 ICU patients that women were less likely to receive invasive mechanical ventilation or renal replacement therapy [25]. However, Peng et al. (2022) found no significant association between sex and the incidence of sepsis-associated acute kidney injury (SA-AKI) in 6463 patients [26].

There are several reasons that underlie these contradictory results. The inadequate adjustment for preexisting comorbidities, disease severity or other silent confounders, as well as selective study populations (medical vs. surgical vs. mixed ICU patients) and different study methods (prospective vs. retrospective vs. database analyses), are only a few reasons to name [27].

However, these inconclusive results do not necessarily disprove the notion that sex affects the pathophysiology of sepsis. Instead, these findings indicate that sex-based variations in sepsis mortality and associated organ dysfunction may not be observed in every patient population or environment. Therefore, studies are needed to elucidate the underlying biological and molecular mechanisms.

Several mechanisms have been proposed to explain the potential protective effect of female sex on sepsis outcomes. There is emerging evidence to suggest that women may have advantageous immunological responses due to the influence of their sexual hormones [28,29]. Contrarily to female sex hormones, which may exhibit protective effects during critical illness, male sex hormones may be deleterious by the effect of a diminished cell-mediated immune response [30]. Specifically, male sex hormones such as androgens have been reported to have a suppressive effect on the cell-mediated immune response [30]. As investigated in animal models, sexual immunomodulation, i.e., testosterone depletion or estrogen supplementation, affects the release of pro-inflammatory and anti-inflammatory cytokines, which are linked to multiorgan failure, and may result in beneficial outcomes in sepsis [30,31]. Additionally, ischemia-reperfusion injury is a common cause of AKI, and experimental studies suggest that sex hormones are involved in the regulation of cellular pathways of this injury and may therefore contribute to AKI susceptibility [26,32,33]. Furthermore, recently investigated biomarkers of mortality, such as free light chains (FLCs) or serum high mobility group box 1 (HMGB1), may serve as important parameters that reflect inflammation and immune dysfunction in CKD and AKI patients [34,35].

There are several limitations to this study that must be taken into consideration when interpreting the results. Due to the prospective and single-center setting of our study cohort, the sample size was relatively small, which may limit the generalizability of the findings. On the other hand, this study comprises a relatively homogenous cohort of sepsis patients from three surgical ICUs using clearly defined inclusion criteria and recording a large number of comorbidities, preexisting medications and clinical follow-up parameters. The findings may not be generalizable to cohorts from mixed or medical ICUs. The extraction and investigation of further biomarkers, cytokines and other organ-specific function parameters, as well as the assessment of long-term outcomes, would have been of great interest and would improve the quality of the present study.

In conclusion, our study found no significant differences in mortality between male and female patients with sepsis and septic shock; however, male patients presented higher organ dysfunction scores and parameters than female patients. These findings are consistent with some previous studies but in contrast to others. Further studies are needed to elucidate the potential underlying mechanisms and therapeutic implications of sex-specific differences in mortality and sepsis-associated organ dysfunction.

## Figures and Tables

**Table 1:** Patient baseline characteristics with regard to sex.

Characteristics	All (n = 737)	Male (n = 484)	Female (n = 253)	*p*-Value
Basic Conditions				
Age [years]	63 ± 15	63 ± 15	63 ± 16	0.7029
Body mass index [kg/m^2^]	28 ± 7	28 ± 7	27 ± 7	0.0062
Severity on Sepsis Onset (Day 1)				
SOFA score	10 ± 4	10 ± 4	9 ± 4	0.0636
APACHE II-score	22 ± 7	22 ± 7	22 ± 7	0.4484
Use of vasopressor [%]	70	69	72	0.4433
Mechanical ventilation [%]	87	87	87	0.9973
Renal replacement therapy [%]	10	11	9	0.5351
Comorbidities [%]				
Arterial hypertension	53	53	53	0.8619
COPD	15	16	12	0.1050
Bronchial asthma	2	2	3	0.3601
Renal dysfunction	10	11	8	0.1156
NIDDM	8	10	5	0.0206
IDDM	10	11	8	0.2232
Chronic liver disease	6	6	4	0.2527
History of myocardial infarction	6	7	4	0.0859
History of stroke	5	6	5	0.5533
History of cancer	14	15	12	0.3671
Medication on Sepsis Onset [%]				
Statins	23	26	19	0.0234
Beta-blocker	37	37	36	0.9559
ACE inhibitor	29	32	23	0.0097
Bronchodilator	10	10	10	0.8481
Diuretics	33	35	30	0.2007
Anticoagulation during the last 6 months	26	27	23	0.2449
Recent Surgical History [%]				
Elective surgery	27	26	30	
Emergency surgery	52	53	50	0.5379
No surgery	21	21	20	
Site of Infection [%]				
Lung	63	67	56	
Abdomen	19	17	22	
Bone or soft tissue	4	3	5	
Surgical wound	2	1	2	0.0838
Urogenital	2	2	4	
Primary bacteremia	6	6	5	
Other	4	4	6	

**Table 2:** Disease severity of patients with sepsis with regard to sex.

Characteristics	All (n = 737)	Male (n = 484)	Female (n = 253)	*p*-Value
Sepsis Severity				
SOFA score	7.2 ± 3.7	7.4 ± 3.6	6.9 ± 3.8	0.0265
Days in septic shock	1 (0, 2)	0 (0, 2)	1 (0, 2)	0.1087
ICU length of stay	21 ± 16	20 ± 15	21 ± 19	0.6748
Hospital length of stay	39 ± 29	40 ± 30	37 ± 27	0.5416
Inflammatory Values				
Leukocytes [1000/µL]	13.2 ± 5	13.1 ± 5.1	13.6 ± 4.9	0.1291
C-reactive protein [mg/L] (n = 380)	150.9 ± 85.7	153.9 ± 86,2	144 ± 84.3	0.3088
Procalcitonin [ng/dL] (n = 657)	1 (0.3, 3.4)	1 (0.3, 3.7)	0.8 (0.3, 2.8)	0.1571
Respiratory Values				
SOFA respiratory subscore	2.0 ± 0.8	2.0 ± 0.8	1.9 ± 0.8	0.0091
Ventilated days	11 ± 8	11 ± 8	11 ± 9	0.2803
Patients with mechanical ventilation [%]	94	94	93	0.4787
Ventilation days/observation days [%]	68 ± 32	69 ± 31	66 ± 32	0.2674
Coagulation				
SOFA coagulation subscore	0 (0, 0.5)	0 (0, 0.5)	0 (0, 0.5)	0.2398
Thrombocytes [1000/µL]	292 ± 150	293 ± 154	290 ± 141	0.7498
Liver Values				
SOFA hepatic subscore	0 (0, 0.4)	0 (0, 0.5)	0 (0, 0.3)	0.1423
Bilirubin [mg/dL]	0.6 (0.4, 1.1)	0.7 (0.5, 1.2)	0.5 (0.4, 0.9)	<0.001
AST [IU/L] (n = 483)	57 (35, 112)	58 (35, 112)	56 (32, 110)	0.8264
ALT [IU/L] (n = 713)	46 (23, 92)	47 (23, 88)	43 (22, 100)	0.3322
Cardiovascular Values				
SOFA cardiovascular subscore	1.6 ± 1	1.6 ± 1	1.7 ± 1.1	0.7080
Vasopressor days	4 (1, 8)	4 (2, 8)	4 (1, 8)	0.9163
Patients with vasopressor treatment [%]	81	81	82	0.6856
Vasopressor days/observation days [%]	29 (11, 57)	29 (11, 56)	31 (11, 57)	0.5938
Central Nervous System				
SOFA central nervous system	2.1 ± 1.1	2.1 ± 1.1	2.1 ± 1.1	0.9434
Glasgow Coma Scale (GCS)	10 ± 3	10 ± 3	10 ± 3	0.9965
Renal Values				
SOFA renal subscore	0.2 (0, 1.2)	0.3 (0, 1.4)	0 (0, 0.8)	<0.001
Creatinine [mg/dL]	1.2 ± 0.9	1.4 ± 1	0.9 ± 0.6	<0.001
Urine output [mL/d]	2904 ± 1341	2878 ± 1334	2954 ± 1357	0.3841
Urine output [mL/kg/d]	1.5 ± 0.8	1.4 ± 0.7	1.7 ± 0.9	<0.001
Dialysed days	0 (0, 0)	0 (0, 0)	0 (0, 0)	0.2947
Patients with renal replacement therapy [%]	22	24	19	0.1078
Dialysis days/observation days [%]	0 (0, 0)	0 (0, 0)	0 (0, 0)	0.2348

**Table 3:** Disease severity of patients with septic shock with regard to sex.

Characteristics	All (n = 373)	Male (n = 237)	Female (n = 136)	*p*-Value
Sepsis Severity				
Sequential Organ Failure Assessment (SOFA)	9 ± 3.9	9.2 ± 3.8	8.7 ± 4	0.1264
Days in septic shock	2 (1, 3)	2 (1, 3)	2 (1, 4)	0.1425
ICU length of stay	24 ± 19	23 ± 16	25 ± 24	0.9666
Hospital length of stay	42 ± 31	43 ± 31	40 ± 31	0.4649
Inflammatory Values				
Leukocytes [1000/µL]	13.9 ± 5.5	13.8 ± 5.7	14.2 ± 5.2	0.3576
C-reactive protein [mg/L] (n = 219)	156 ± 79	160 ± 79	147 ± 78	0.2204
Procalcitonin [ng/dL] (n = 352)	1.8 (0.7, 5.9)	2.2 (0.9, 6.1)	1.4 (0.5, 5.4)	0.0804
Respiratory Values				
SOFA respiratory subscore	2.2 ± 0.7	2.3 ± 0.8	2.1 ± 0.7	0.1089
Ventilated days	13 ± 9	13 ± 9	13 ± 9	0.9130
Patients with mechanical ventilation [%]	97	97	96	0.8788
Ventilation days/observation days [%]	74 ± 29	75 ± 29	73 ± 30	0.8123
Coagulation				
SOFA coagulation subscore	0.2 (0, 0.9)	0.2 (0, 1)	0.3 (0, 0.9)	0.9773
Thrombocytes [1000/µL]	251 ± 142	257 ± 154	240 ± 118	0.7232
Liver Values				
SOFA hepatic subscore	0.1 (0, 1)	0.1 (0, 0.9)	0.1 (0, 1)	0.6701
Bilirubin [mg/dL]	0.8 (0.5, 1.6)	0.8 (0.5, 1.6)	0.7 (0.4, 1.7)	0.1118
AST (GOT) [IU/L] (n = 291)	69 (40, 140)	71 (41, 160)	67 (38, 121)	0.6234
ALT (GPT) [IU/L] (n = 362)	44 (22, 101)	49 (25, 98)	37 (19, 106)	0.0964
Cardiovascular Values				
SOFA cardiovascular subscore	2.1 ± 1	2.1 ± 1	2.1 ± 1	0.7594
Vasopressor days	8 ± 6	8 ± 6	9 ± 7	0.7382
Patients with vasopressor treatment [%]	100	100	100	1.0
Vasopressor days/observation days [%]	52 ± 30	51 ± 30	53 ± 30	0.5564
Central Nervous System				
SOFA central nervous system	2.3 ± 1	2.3 ± 1	2.3 ± 1	0.8255
Glasgow Coma Scale (GCS)	9 ± 3	9 ± 3	9 ± 3	0.9277
Renal Values				
SOFA renal subscore	0.6 (0.1, 2)	0.8 (0.2, 2.3)	0.3 (0, 1.3)	0.0021
Creatinine [mg/dL]	1.4 ± 1	1.6 ± 1	1.1 ± 0.7	<0.001
Urine output [mL/d]	2661 ± 1540	2661 ± 1549	2661 ± 1531	0.9924
Urine output [mL/kg/d]	1.4 ± 0.8	1.3 ± 0.8	1.5 ± 0.9	0.0326
Dialysed days	0 (0, 4)	0 (0, 4)	0 (0, 3)	0.1682
Patients with renal replacement therapy [%]	39	43	32	0.0503
Dialysis days/observation days [%]	0 (0, 28)	0 (0, 33)	0 (0, 18)	0.1027

**Table 4:** Multivariate Cox proportional hazards regression analysis for patients with sepsis.

	28-Day Mortality			90-Day Mortality		
Variables	HR	95%-CI	*p*-Value	HR	95%-CI	*p*-Value
Male sex	1.25	0.89–1.76	0.2012	1.09	0.83–1.44	0.5236
Age	1.04	1.02–1.05	<0.001	1.04	1.02–1.05	<0.001
BMI	0.98	0.95–1.01	0.1439	1	0.97–1.02	0.7119
NIDDM	1.13	0.64–2.02	0.6702	0.99	0.64–1.55	0.9748
Statins	0.73	0.49–1.08	0.1120	0.84	0.62–1.16	0.2897
ACE inhibitor	1.05	0.73–1.5	0.7996	1.04	0.78–1.4	0.7868

**Table 5:** Multivariate Cox proportional hazards regression analysis for patients in septic shock.

	28-Day Mortality			90-Day Mortality		
Variables	HR	95%-CI	*p*-Value	HR	95%-CI	*p*-Value
Sex	1.25	0.84–1.87	0.2699	1.15	0.82–1.6	0.4142
Age	1.03	1.01–1.04	<0.001	1.03	1.01–1.04	<0.001
BMI	0.97	0.93–1	0.0753	0.99	0.97–1.01	0.3743
NIDDM	0.94	0.5–1.78	0.8483	1	0.58–1.73	0.9893
Statins	0.79	0.49–1.28	0.3437	0.92	0.62–1.36	0.6684
ACE inhibitor	1.11	0.71–1.73	0.6454	1.12	0.78–1.62	0.5665

## Data Availability

The datasets generated and/or analyzed during the current study are available from the corresponding author upon reasonable request.

## References

[1] Singer M., Deutschman C.S., Seymour C.W., Shankar-Hari M., Annane D., Bauer M., Bellomo R., Bernard G.R., Chiche J.-D., Coopersmith C.M. (2016). The Third International Consensus Definitions for Sepsis and Septic Shock (Sepsis-3). JAMA.

[2] Markwart R., Saito H., Harder T., Tomczyk S., Cassini A., Fleischmann-Struzek C., Reichert F., Eckmanns T., Allegranzi B. (2020). Epidemiology and Burden of Sepsis Acquired in Hospitals and Intensive Care Units: A Systematic Review and Meta-Analysis. Intensive Care Med..

[3] Fleischmann-Struzek C., Rose N., Freytag A., Spoden M., Prescott H.C., Schettler A., Wedekind L., Ditscheid B., Storch J., Born S. (2021). Epidemiology and Costs of Postsepsis Morbidity, Nursing Care Dependency, and Mortality in Germany, 2013 to 2017. JAMA Netw. Open.

[4] Kumar G., Kumar N., Taneja A., Kaleekal T., Tarima S., McGinley E., Jimenez E., Mohan A., Khan R.A., Whittle J. (2011). Nationwide Trends of Severe Sepsis in the 21st Century (2000–2007). Chest.

[5] Gaieski D.F., Edwards J.M., Kallan M.J., Carr B.G. (2013). Benchmarking the Incidence and Mortality of Severe Sepsis in the United States. Crit. Care Med..

[6] Angus D.C., Linde-Zwirble W.T., Lidicker J., Clermont G., Carcillo J., Pinsky M.R. (2001). Epidemiology of Severe Sepsis in the United States: Analysis of Incidence, Outcome, and Associated Costs of Care. Crit. Care Med..

[7] Shankar-Hari M., Phillips G.S., Levy M.L., Seymour C.W., Liu V.X., Deutschman C.S., Angus D.C., Rubenfeld G.D., Singer M., Sepsis Definitions Task Force (2016). Developing a New Definition and Assessing New Clinical Criteria for Septic Shock: For the Third International Consensus Definitions for Sepsis and Septic Shock (Sepsis-3). JAMA.

[8] Adrie C., Azoulay E., Francais A., Clec’h C., Darques L., Schwebel C., Nakache D., Jamali S., Goldgran-Toledano D., Garrouste-Orgeas M. (2007). Influence of Gender on the Outcome of Severe Sepsis: A Reappraisal. Chest.

[9] Colbert J.F., Traystman R.J., Poisson S.N., Herson P.S., Ginde A.A. (2016). Sex-Related Differences in the Risk of Hospital-Acquired Sepsis and Pneumonia Post Acute Ischemic Stroke. J. Stroke Cerebrovasc. Dis..

[10] Xu J., Tong L., Yao J., Guo Z., Lui K.Y., Hu X., Cao L., Zhu Y., Huang F., Guan X. (2019). Association of Sex With Clinical Outcome in Critically Ill Sepsis Patients: A Retrospective Analysis of the Large Clinical Database MIMIC-III. Shock.

[11] Sakr Y., Elia C., Mascia L., Barberis B., Cardellino S., Livigni S., Fiore G., Filippini C., Ranieri V.M. (2013). The Influence of Gender on the Epidemiology of and Outcome from Severe Sepsis. Crit. Care.

[12] Crabtree T.D., Pelletier S.J., Gleason T.G., Pruett T.L., Sawyer R.G. (1999). Gender-Dependent Differences in Outcome after the Treatment of Infection in Hospitalized Patients. JAMA.

[13] Pietropaoli A.P., Glance L.G., Oakes D., Fisher S.G. (2010). Gender Differences in Mortality in Patients with Severe Sepsis or Septic Shock. Gender Med..

[14] von Elm E., Altman D.G., Egger M., Pocock S.J., Gøtzsche P.C., Vandenbroucke J.P., STROBE Initiative (2007). The Strengthening the Reporting of Observational Studies in Epidemiology (STROBE) Statement: Guidelines for Reporting Observational Studies. Lancet.

[15] Levy M.M., Fink M.P., Marshall J.C., Abraham E., Angus D., Cook D., Cohen J., Opal S.M., Vincent J.-L., Ramsay G. (2003). 2001 SCCM/ESICM/ACCP/ATS/SIS International Sepsis Definitions Conference. Crit. Care Med..

[16] Mewes C., Böhnke C., Alexander T., Büttner B., Hinz J., Popov A.-F., Ghadimi M., Beißbarth T., Raddatz D., Meissner K. (2019). Favorable 90-Day Mortality in Obese Caucasian Patients with Septic Shock According to the Sepsis-3 Definition. J. Clin. Med..

[17] Mewes C., Büttner B., Hinz J., Alpert A., Popov A.-F., Ghadimi M., Beissbarth T., Tzvetkov M., Jensen O., Runzheimer J. (2019). CTLA-4 Genetic Variants Predict Survival in Patients with Sepsis. J. Clin. Med..

[18] Mewes C., Büttner B., Hinz J., Alpert A., Popov A.F., Ghadimi M., Beissbarth T., Tzvetkov M., Shen-Orr S., Bergmann I. (2018). The CTLA-4 Rs231775 GG Genotype Is Associated with Favorable 90-Day Survival in Caucasian Patients with Sepsis. Sci. Rep..

[19] Mewes C., Alexander T., Büttner B., Hinz J., Alpert A., Popov A.-F., Ghadimi M., Beißbarth T., Tzvetkov M., Grade M. (2020). TIM-3 Genetic Variants Are Associated with Altered Clinical Outcome and Susceptibility to Gram-Positive Infections in Patients with Sepsis. Int. J. Mol. Sci..

[20] Mewes C., Alexander T., Büttner B., Hinz J., Alpert A., Popov A.-F., Beißbarth T., Tzvetkov M., Grade M., Quintel M. (2021). Effect of the Lymphocyte Activation Gene 3 Polymorphism Rs951818 on Mortality and Disease Progression in Patients with Sepsis-A Prospective Genetic Association Study. J. Clin. Med..

[21] Hinz J., Büttner B., Kriesel F., Steinau M., Frederik Popov A., Ghadimi M., Beissbarth T., Tzvetkov M., Bergmann I., Mansur A. (2017). The FER Rs4957796 TT Genotype Is Associated with Unfavorable 90-Day Survival in Caucasian Patients with Severe ARDS Due to Pneumonia. Sci. Rep..

[22] Madsen T.E., Simmons J., Choo E.K., Portelli D., McGregor A.J., Napoli A.M. (2014). The DISPARITY Study: Do Gender Differences Exist in Surviving Sepsis Campaign Resuscitation Bundle Completion, Completion of Individual Bundle Elements, or Sepsis Mortality?. J. Crit. Care.

[23] van Vught L.A., Scicluna B.P., Wiewel M.A., Hoogendijk A.J., Klein Klouwenberg P.M.C., Ong D.S.Y., Cremer O.L., Horn J., Franitza M., Toliat M.R. (2017). Association of Gender With Outcome and Host Response in Critically Ill Sepsis Patients. Crit. Care Med..

[24] Thompson K.J., Finfer S.R., Woodward M., Leong R.N.F., Liu B. (2022). Sex Differences in Sepsis Hospitalisations and Outcomes in Older Women and Men: A Prospective Cohort Study. J. Infect..

[25] Modra L.J., Higgins A.M., Abeygunawardana V.S., Vithanage R.N., Bailey M.J., Bellomo R. (2022). Sex Differences in Treatment of Adult Intensive Care Patients: A Systematic Review and Meta-Analysis. Crit. Care Med..

[26] Peng J., Tang R., Yu Q., Wang D., Qi D. (2022). No Sex Differences in the Incidence, Risk Factors and Clinical Impact of Acute Kidney Injury in Critically Ill Patients with Sepsis. Front. Immunol..

[27] Lakbar I., Einav S., Lalevée N., Martin-Loeches I., Pastene B., Leone M. (2023). Interactions between Gender and Sepsis—Implications for the Future. Microorganisms.

[28] Sperry J.L., Nathens A.B., Frankel H.L., Vanek S.L., Moore E.E., Maier R.V., Minei J.P., Inflammation and the Host Response to Injury Investigators (2008). Characterization of the Gender Dimorphism after Injury and Hemorrhagic Shock: Are Hormonal Differences Responsible?. Crit. Care Med..

[29] Klein S.L., Jedlicka A., Pekosz A. (2010). The Xs and Y of Immune Responses to Viral Vaccines. Lancet Infect. Dis..

[30] Angele M.K., Pratschke S., Hubbard W.J., Chaudry I.H. (2014). Gender Differences in Sepsis: Cardiovascular and Immunological Aspects. Virulence.

[31] Diodato M.D., Knöferl M.W., Schwacha M.G., Bland K.I., Chaudry I.H. (2001). Gender Differences in the Inflammatory Response and Survival Following Haemorrhage and Subsequent Sepsis. Cytokine.

[32] Viñas J.L., Porter C.J., Douvris A., Spence M., Gutsol A., Zimpelmann J.A., Tailor K., Campbell P.A., Burns K.D. (2020). Sex Diversity in Proximal Tubule and Endothelial Gene Expression in Mice with Ischemic Acute Kidney Injury. Clin. Sci..

[33] Hutchens M.P., Fujiyoshi T., Komers R., Herson P.S., Anderson S. (2012). Estrogen Protects Renal Endothelial Barrier Function from Ischemia-Reperfusion in Vitro and in Vivo. Am. J. Physiol. Renal Physiol..

[34] Lacquaniti A., Campo S., Falliti G., Caruso D., Gargano R., Giunta E., Monardo P. (2022). Free Light Chains, High Mobility Group Box 1, and Mortality in Hemodialysis Patients. J. Clin. Med..

[35] Campo S., Lacquaniti A., Trombetta D., Smeriglio A., Monardo P. (2022). Immune System Dysfunction and Inflammation in Hemodialysis Patients: Two Sides of the Same Coin. J. Clin. Med..

